# Trust in institutions and misinformation susceptibility both independently explain vaccine skepticism

**DOI:** 10.1038/s41598-025-21452-1

**Published:** 2025-10-28

**Authors:** Thom Roozenbeek, Caspar van den Berg, Mattijs S. Lambooij, Sander van der Linden, Rakoen Maertens, José A. Ferreira, Mart van Dijk, Jon Roozenbeek

**Affiliations:** 1 Department of Global and Local Governance, University of Groningen, 8911 CE Leeuwarden, The Netherlands; 2 Erasmus School of Health Policy and Management, 3062 PA Rotterdam, The Netherlands; 3 Department of Psychology, University of Cambridge, Cambridge, CB2 3EB UK; 4 Department of Experimental Psychology, University of Oxford, Oxford, OX2 6GG UK; 5 National Institute for Public Health and the Environment, Bilthoven, 3721 MA The Netherlands; 6 Department of War Studies, King’s College London, London, WC2R 2LS UK

**Keywords:** Institutional trust, Misinformation, Vaccine skepticism, Vaccine hesitancy, Psychology, Health care, Health policy, Public health

## Abstract

Herd immunity for preventable childhood infectious diseases such as measles and mumps has come under threat in numerous countries, due to declining vaccination rates. This decline underscores the urgent need to understand the underlying mechanisms of vaccine skepticism. Institutional (dis)trust and belief in (vaccine) misinformation have been proposed as important factors, but their interconnectedness and potential mutual influence have remained elusive. Importantly, higher trust has been hypothesized to serve as a “buffer” against the adverse effects of misinformation belief. In this preregistered study (*N* = 1356, probability sample of Dutch households), we address these questions using validated, high-quality measures of vaccine attitudes, trust, and misinformation susceptibility. We find that specific trust in the government with respect to vaccinations is a much stronger predictor of vaccine skepticism than general trust in institutions. Moreover, susceptibility to misinformation is significantly associated with vaccine skepticism under all model specifications. Contrary to expectations, we find no evidence for the “buffer” hypothesis, suggesting that, while correlated, misinformation susceptibility and trust are distinctly related to vaccine skepticism.

**Supplementary Information:**

The online version contains supplementary material available at 10.1038/s41598-025-21452-1.

## Introduction

Despite the overwhelming consensus among experts on the importance of vaccines, a global study across 149 countries revealed that a significant portion of the population remains vaccine-hesitant, doubting their importance, safety, or effectiveness^1^. In the Netherlands, findings show a decline of roughly 2–5% in coverage of childhood vaccinations between 2022 and 2023, approaching or surpassing the lower threshold for herd immunity for infectious diseases in multiple regions, increasing the risk of the spread of infectious diseases such as measles and chickenpox^2^. Comparable patterns have been observed in other European countries, though the specific trends vary depending on the country and the vaccine in question. Understanding the underlying mechanisms of declining vaccination coverage is of key importance to scientists and policymakers. Recent research has emphasized the role of both (a lack of) trust in institutions and belief in (vaccine) misinformation, with both having been shown to be strongly associated with vaccine skepticism^3,4^. However, an important knowledge gap remains regarding their interconnectedness and potential mutual influence. Moreover, trust has been hypothesized to serve as a “buffer” for the adverse effects of misinformation belief^5^.

Although misinformation has been linked to various visibly negative societal outcomes, such as lower compliance with public health guidelines^6^, lower support for climate change^7^, there is an ongoing scientific debate whether misinformation is the primary *cause* of such negative societal outcomes, or whether it is a secondary *symptom* of more important underlying issues (e.g., lack of trust). On the one hand, scholars call for policies and interventions that address the spread of and susceptibility to misinformation, and note that there is a clear link between misinformation belief and a variety of adverse attitudes, beliefs, and behaviors^8,9^. On the other hand, researchers have warned against a misinformation “moral panic”, arguing that focusing on reducing exposure to or belief in misinformation misses the mark^10,11^. This view proposes that problematic outcomes such as vaccine hesitancy and climate change denial are caused by deeper socio-economic and political issues such as an erosion of trust in institutions or political polarization. Interventions should therefore not (only) address misinformation as such, but rather the ‘root’ of these problems. While we cannot disentangle cause and effect given the correlational research design of our study, this debate prompts the need to compare the relative influence of trust and misinformation on vaccine skepticism.

There are good reasons to expect an effect of (susceptibility to) misinformation on vaccine skepticism. For example, there is a widespread concern amongst parents that vaccinations could cause autism, informed in large part by misinformation such as the infamous retracted *Lancet* paper by Andrew Wakefield^12,13^. Misinformation about a supposed link between the MMR vaccine and autism has been associated with a significant decline in vaccination coverage in the UK^14^. Conspiracy theories about COVID-19 vaccines have also been linked to increased vaccine hesitancy and reduced vaccination intentions^15^. Other studies have shown that misinformation can causally alter beliefs and behaviors^16,17^. One study in the UK showed that susceptibility to misinformation predicted reduced uptake of COVID-19 vaccinations^4^, although causal evidence remains limited. Moreover, it remains unknown whether similar results are found for childhood vaccinations, which is important because COVID-19 vaccines have been more subject to political debate than childhood vaccines^18^.

Susceptibility to misinformation may not only affect vaccine skepticism directly but also negatively affect trust in institutions and vice versa^19^. Researchers have warned of a “*self-reinforcing cycle in which media-skeptical citizens turn to content that further reinforces their doubts*”^20^. However, the direction of this relationship has not yet been fully established. On the one hand, some studies suggest that higher trust decreases misinformation belief, whereas lower trust increases it. For example, higher trust in science was strongly associated with a lower belief in COVID-19 misinformation in a study across 5 countries: Mexico, Spain, Ireland, the UK and the USA^6^. Another study showed that higher trust in official governmental information was associated with decreased belief in false and misleading information about COVID-19, whereas trust in news from social media outlets contributed to increased misinformation belief^21^. On the other hand, studies suggest that misinformation affects distrust in governmental, political, and media institutions^22–24^. Indeed, an oft-mentioned concern about misinformation is that it may erode trust in institutions^25^. Seeing misinformation that contradicts with reliable news sources, such as official information, may boost doubts about the truthfulness and accuracy of official information and thereby reduce trust^26,27^. We therefore hypothesize that susceptibility to misinformation is negatively associated with institutional trust **(H1).**

It is widely acknowledged that distrust is a key predictor of vaccine hesitancy^28^ and is associated with lower vaccine uptake^29^. However, there is an ongoing debate regarding how trust should be measured. Some argue it should be seen as a unidimensional construct^30^, whereas others urge scientists to be wary of ‘generalizations’ when measuring trust, and instead emphasize the need to use *specific* rather than general measures (see “Materials and methods” section)^33^. Overall, we hypothesize that susceptibility to misinformation and institutional trust are significant predictors of vaccine skepticism **(H2).**

### The “buffer” hypothesis

To our knowledge, although hypothesized^19^, no studies have empirically evaluated the interconnectedness between misinformation and trust and whether one of these two factors cancels out the effect of the other on societal outcomes. Research in other issue domains (e.g., trust in media) suggests that trust could function as a “buffer” against misinformation, in the sense that it could strengthen resistance to misinformation. For instance, people are more likely to make use of credible news sources if they trust (mainstream) news media and official information, and are less likely to perceive misinformation as true and are less likely to share it^32,33^. People rely on trust in their decisions to follow governmental or scientific recommendations for vaccinating their children, even if their knowledge about vaccines is limited, which is the case for the majority of the population^34^. In other words, people with high levels of trust may evaluate institutional intentions and efforts more positively than those with low trust, with trust thereby acting as a buffer against misinformation^5^. This notion is supported by findings showing that social media exposure is positively associated with misbeliefs about HPV vaccinations^35^. In this study, the negative association of HPV related misbeliefs with vaccination policy support was larger for those who had low levels of trust, whereas people with high levels of trust did not show this effect. The negative effect of misinformation on vaccine attitudes and behaviors may therefore be weaker among those with high levels of trust. Additionally, a “flooring effect” could occur for those who have low trust to begin with: because people with low levels of trust are more likely to have negative attitudes regarding vaccines and therefore avoid vaccinations, it may be that there is little room for misinformation to have an additional effect. Following this logic, we hypothesize that the association between misinformation susceptibility and vaccine skepticism is weaker for people with high institutional trust than for people with low institutional trust. In other words, susceptibility to misinformation strengthens the relationship between low institutional trust and vaccine skepticism (**H3**).

**Fig. 1:**
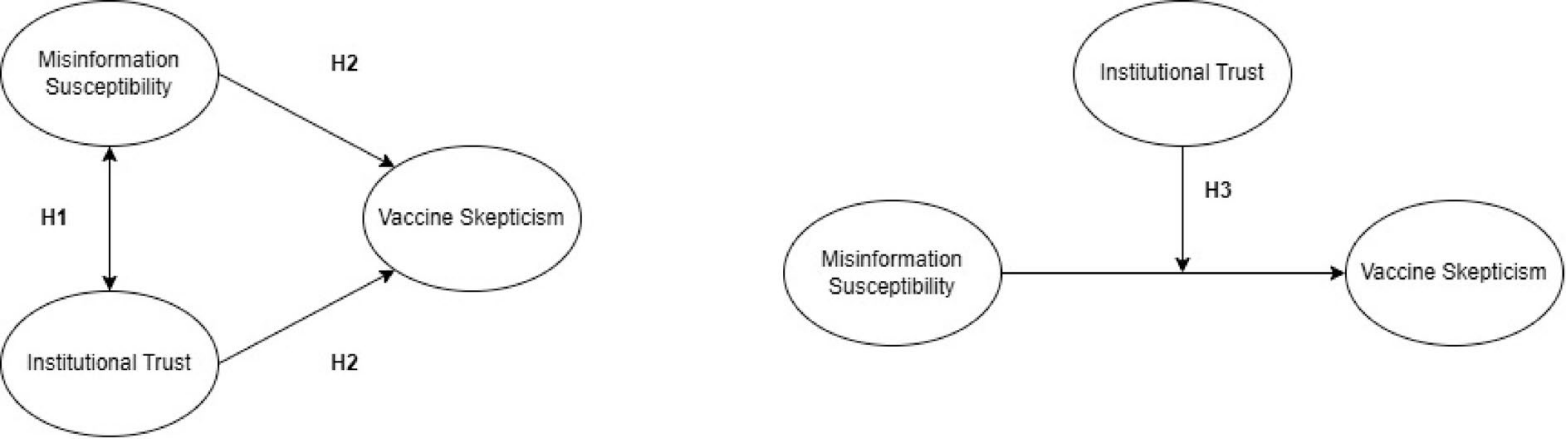
Conceptual models. *Note* H1 tests whether misinformation and institutional trust are associated. H2 tests whether both institutional trust and misinformation susceptibility predict vaccine skepticism variables. H3 tests whether trust moderates the relationship between misinformation susceptibility and vaccine skepticism variables (buffer hypothesis).

The conceptual model in Fig. 1 visually summarizes the hypotheses. While our analyses are correlational in nature, the models tested are informed by *hypothesized* causal relationships based on prior research mentioned above. These directional assumptions guide our hypotheses and model structure but we emphasize that we do not interpret results as evidence of causality.

## Results

To address the above questions, we collected a total of 1,356 responses through the Dutch LISS (Longitudinal Internet Studies for the Social Sciences) panel. This panel collects a probability-weighted sample of the Dutch population, collecting responses for a variety of questionnaires, including on demographics, politics, trust, health and more; see Appendix 1 for sample descriptives. For this study, we administered a battery of validated measures on trust, vaccine-related attitudes and behaviors and misinformation susceptibility (specifically a translated, 12-item version of the Misinformation Susceptibility Test or MIST^36^). For our various measures of institutional trust we conducted a series of Exploratory and Confirmatory Factor Analyses (see “Materials and methods” section). When measuring trust in several institutions, we found that institutional trust can be broken down into two distinct scales. Institutional trust **A** broadly includes political institutions such as parliament and politicians. Institutional trust **B** primarily includes health-related institutions such as the National Institute of Public Health and general practitioners (GPs). Finally, we also included a more specific scale that measures individuals’ trust in government specifically with respect to vaccinations (specific institutional trust). For more details regarding the sample and measures, including deviations from the preregistration (https://aspredicted.org/ei25q.pdf), see “Materials and methods” section and Appendix 2.

### Institutional trust and misinformation susceptibility (H1)

To assess the correlation between the trust measures and misinformation susceptibility, we conducted non-parametric permutation tests and correcting for multiple testing by controlling for the false discovery rate^37^. In order to try to test each association as specifically as possible, the tests were carried out by using a bootstrap method and by controlling for potential confounders, namely gender, age, income, education and origin (i.e., Dutch origin versus individuals with a migration background, including both migrants and children of migrants). Statistical significance was determined using a nominal FDR threshold of 5%, meaning that among 20 reported discoveries, no more than one is expected to be a false positive on average. Table 1 shows the pairwise associations detected in this way between the trust measures and veracity discernment (our operationalization for misinformation susceptibility, see “Materials and methods” section). The results show significant small to modest correlations. Veracity discernment is positively associated with trust in political institutions (institutional trust A), trust in health institutions (institutional trust B), as well as for specific trust in government with respect to vaccinations (specific trust). In other words, for all measures of institutional trust, individuals with higher misinformation susceptibility tend to have lower trust levels, in support of **H1**. We note that we preregistered to conduct regression models for this hypothesis, but noticed post-preregistration that permutation tests would be more prudent. Our preregistered analyses are presented in Appendix 3.1, showing highly comparable results: also in the linear regression models, small to moderate between trust and misinformation susceptibility are observed, also after controlling for confounders.

**Table 1:** Association table between veracity discernment and trust.

Variable	Variable	*p*	FDR	Correlation coefficient	CI (95%)
Veracity discernment	Institutional trust A	< .001	< 0.05	.14	(0.07, 0.21)
	Institutional trust B	< .001	< 0.05	.28	(0.24, 0.37)
	Specific Trust	< .001	< 0.05	.32	(0.27, 0,39)

To assess the correlations as specifically as possible, we controlled for confounding factors (sex, age, education, income and origin).

### Institutional trust, misinformation susceptibility, and vaccine skepticism (H2)

Similar to H1, the association of each explanatory variable with each of the outcome variables was assessed by non-parametric permutation tests and correcting for multiple testing by controlling for the false discovery rate^37^. The results (Table 2) show that the strongest correlation coefficients are found between the dependent variables and both specific trust and institutional trust B (i.e., trust in health institutions). Because vaccine concern and hesitancy reflect psychological states rather than actual behavior, we used vaccine refusal as a dependent variable to give an indication of behavior. We included multiple variables for vaccine skepticism (i.e., vaccine concern, hesitancy, and refusal, see “Materials and methods” section).

**Table 2:** Associations between vaccine skepticism measures, veracity discernment and trust.

Dependent variable	Independent variable	*p*	FDR	Correlation coefficient	CI (95%)
Vaccine concern^1^	Veracity discernment	< .001	< .05	− .290	(− .372, − .256)
	Specific trust	< .001	< .05	− .576	(− .644, − .533)
	Institutional trust A	< .001	< .05	− .186	(− .267, − .128)
	Institutional trust B	< .001	< .05	− .503	(− .581, − .457)
Vaccine hesitancy^2^	Veracity discernment	< .001	< .05	− .171	(− .277, − .106)
	Specific trust	< .001	< .05	− .388	(− .486, − .301)
	Institutional trust A	.143	NS	NS	NS
	Institutional trust B	< .001	< .05	− .322	(− .428, − .247)
Vaccine refusal^2^	Veracity discernment	.288	NS	NS	NS
	Specific trust	< .001	< .05	.359	(.241, .503)
	Institutional trust A	.001	< .05	.186	(.063, .352)
	Institutional trust B	< .001	< .05	.346	(.263, .517)

We controlled for potential confounders (i.e., age, gender, education, income, origin). Vaccine concern^1^ was measured for all respondents (n = 1356). Hesitancy and refusal^2^ were measured only for respondents who indicated to have children (n = 624). Abbreviations: NS = not significant. FDR = false discovery rate. CI = confidence interval. Out of 624 respondents with children, only 22 parents indicated their child received no vaccinations for which they have been invited so far, and 63 indicated ‘some’ vaccinations. The large majority of respondents with children (n = 539) indicated that their youngest child received all vaccinations they have been invited for so far, which is similar to percentages observed on a national level^2^. The correlation coefficients of vaccine refusal have positive values because of the way this variable is coded (see “Materials and methods” section).

**Fig. 2:**
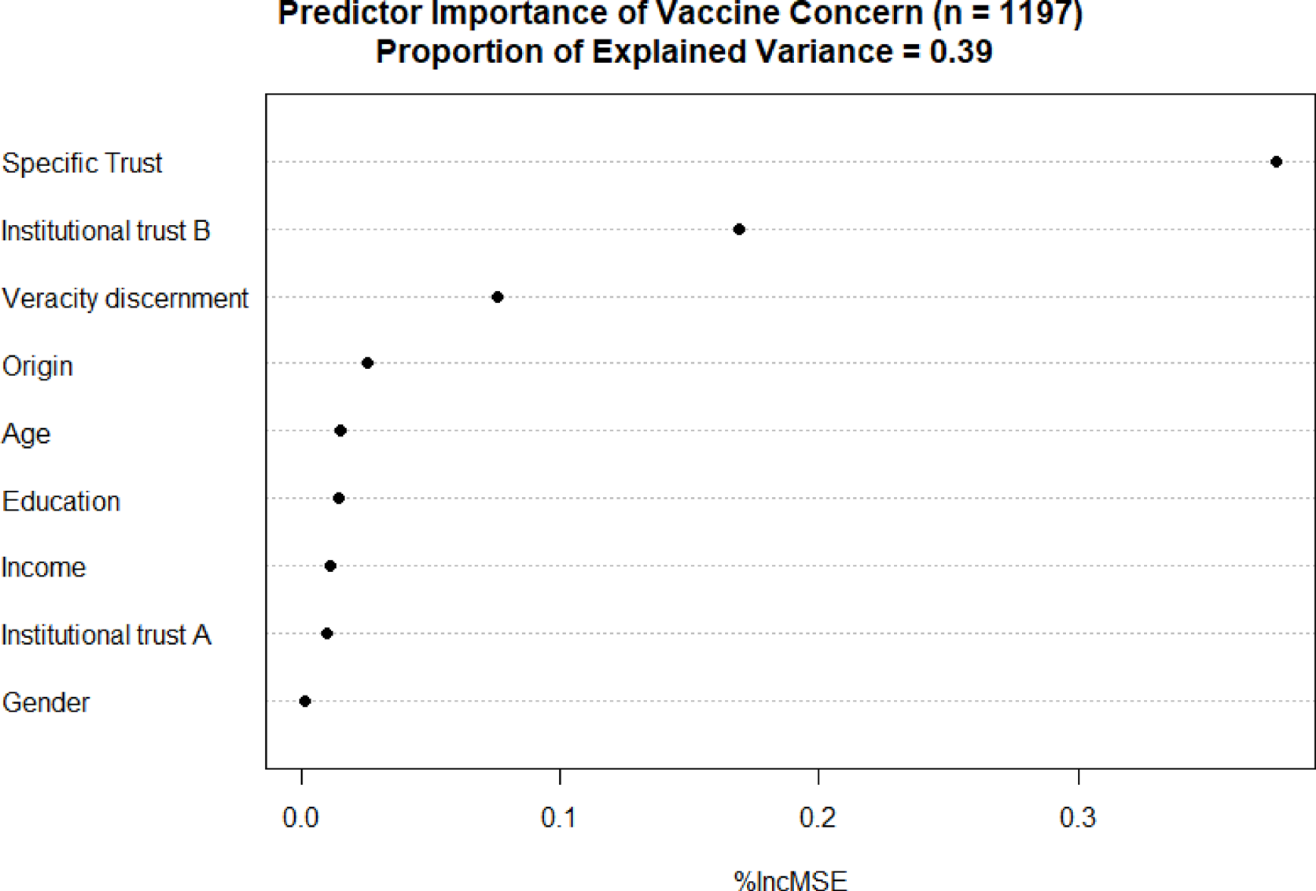
Variable importance ranking of vaccine concern. *Note* The RF model explains 39% of the variance. The model achieved a Mean Absolute Error (MAE) of 0.53, which suggests that the model provides a reasonably accurate approximation of vaccine concern. Note: the *n* of this model is 1197, which is lower than the full dataset (*n* = 1356), because the forest model was trained using only respondents who provided complete data for all variables included in the model.

To complement the association analyses we studied whether and how the various trust measures, veracity discernment, and the demographic variables predict vaccine concern and hesitancy. We applied a Random Forest analysis (RF)^38,39^ for this purpose. This is a machine learning technique for both regression and classification that utilizes a nonparametric ensemble of decision trees. We considered this an appropriate method because of (potentially) intercorrelated independent variables, as well as potential non-linear relationships between the independent and dependent variables. We note that this method was not preregistered, as we noticed post-preregistration that it would be more prudent. However, our preregistered analyses show highly similar results (see Fig. S5 in Appendix 3.2). The overall performance of the prediction analysis was assessed in terms of the percentage explained variance (0.39). The relative weights of the independent variables in predicting vaccine concern were assessed by ranking their ‘importance’. Variable importance was quantified in terms of the percentage increase in mean-squared error (MSE) when the effect of that variable was removed. The RF model (Fig. 2) shows that specific trust, institutional trust B and Veracity Discernment are the strongest predictors, reinforcing the findings from association analyses presented in Table 2. A similar pattern emerged for the RF model for vaccine hesitancy, where specific trust is also the most important predictor (see Fig. S6 in Appendix 3.2).

### Buffer hypothesis (H3)

**Fig. 3:**
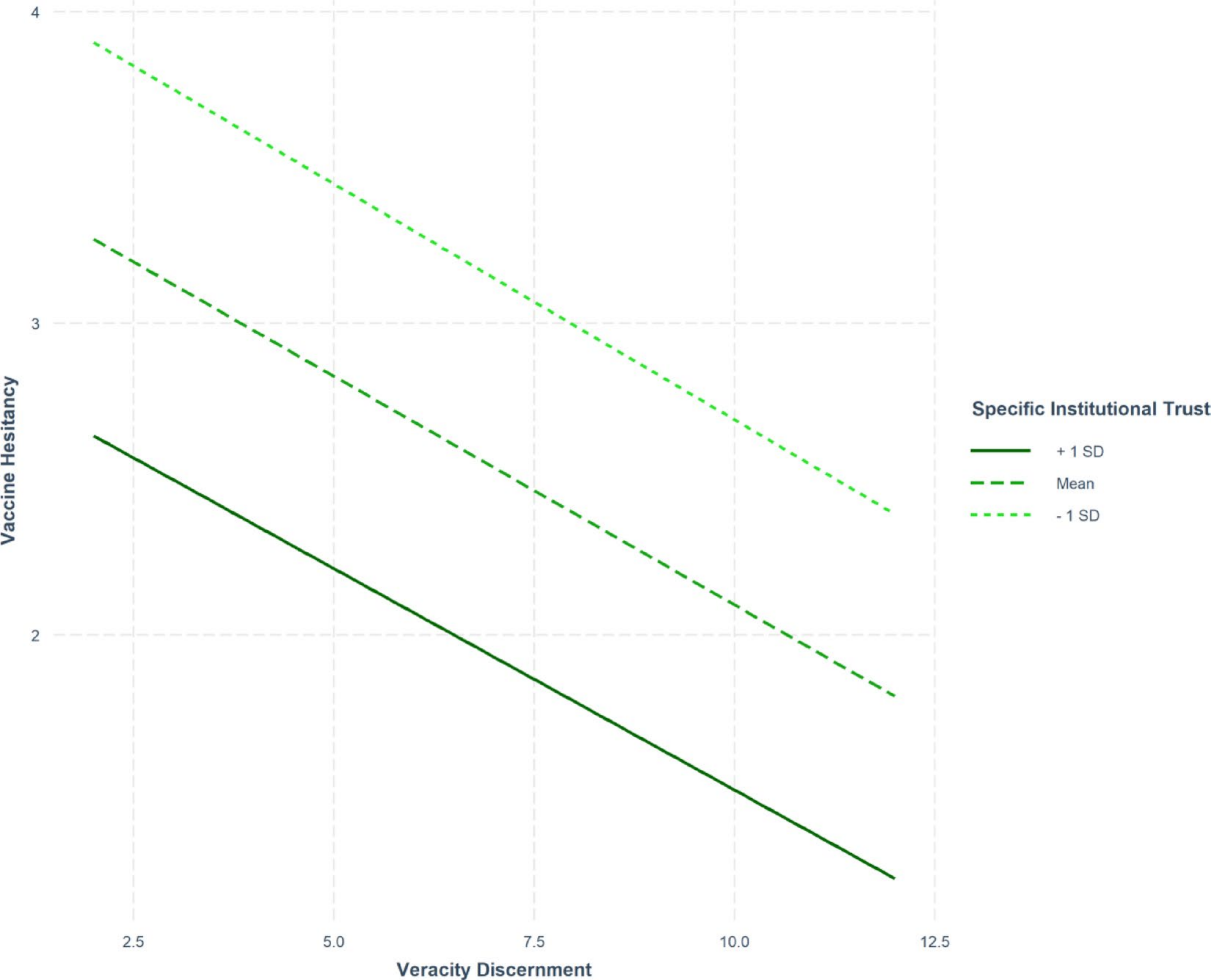
Interaction between veracity discernment (as measured by the Misinformation Susceptibility Test) and vaccine hesitancy for different levels of specific institutional trust (in the government with respect to vaccinations). *Note* The interaction is not significant (*p* = .555). No significant interaction effects are observed under different model specifications (e.g., using different measures of trust or vaccine hesitancy or concern).

For the moderation analyses, we fitted a series of preregistered linear regression models predicting our two main dependent variables (vaccine concern and hesitancy), looking at the interaction terms between veracity discernment and our three measures of institutional trust, and the same demographic variables as covariates. We find no significant interaction effects in any of our models (all *p* values > 0.125 see Appendix 3.3). This indicates that trust does not significantly moderate the relationship between misinformation susceptibility and vaccine hesitancy, and that we find no evidence of “flooring effects” for people with lower levels of trust. Figure 3 shows an illustrative example (see for the full analyses Appendix 3.1 and 3.2). Overall, our data do not support the buffer hypothesis or **H3**.

## Discussion

In this preregistered survey study of a probability-weighted sample of 1,356 residents of the Netherlands, we tested whether vaccine skepticism is predicted by different measures of (institutional) trust and misinformation susceptibility, and whether high trust can serve as a “buffer” against the adverse effects of misinformation on vaccine attitudes.

We find that institutional (dis)trust is a robust predictor of vaccine skepticism. Individuals with low trust were substantially more likely to express vaccine concern, hesitancy, or even refuse to vaccinate their children, also after controlling for socio-demographic characteristics. We find that a decrease in *generalized* trust in government may not be the prime driver of the observed rise in vaccine skepticism and diminishing childhood vaccination rates. Rather, *specific* trust in the government when it comes to vaccinations (a compound measure which includes questions such as “*the government acts in the interests of citizens when it comes to vaccination*s”) was by far the strongest predictor of vaccine skepticism in all model specifications. We therefore argue that individuals distinguish between their opinion about the government as a whole, and their (lack of) trust in the government’s childhood vaccination programs. Institutional credibility is critical to fostering public compliance with health measures such as vaccinations. Scholars have argued that a certain level of skepticism towards government is beneficial, and even a fundamental right of people living in democracies^40^. In the Netherlands, a meaningful part of the population is pessimistic about the direction the country is heading, and has no confidence in the problem-solving capacity of government and the intentions of decision-makers^41^. Confidence is dependent of government performance: it often fluctuates with major developments and is based on satisfaction with performance of the various levels and branches of government^42^. Our findings are therefore encouraging from a policy perspective: to increase childhood vaccination coverage, governments may seek to (re)gain trust in their vaccination policy specifically, but they do not necessarily need to reduce general antipathy toward government and its many institutions (which is a much taller order).

Second, across all models, higher misinformation susceptibility predicted vaccine skepticism, including after controlling for demographics and, importantly, different measures of institutional trust. This finding contributes to the ongoing debate over whether misinformation is a symptom or a cause of societal problems such as diminishing vaccination rates^19,43^. Briefly put, we find that some measures of trust are indeed a stronger predictor of vaccine attitudes and behaviors than misinformation susceptibility, which supports the idea that underlying societal factors (not only institutional trust but also polarization, political fragmentation, etc.) “matter more” than individual-level exposure to and belief in misinformation (though it is important to consider that low trust may in itself be a consequence of prior misinformation exposure, a reciprocal causal process we cannot disentangle here). At the same time, misinformation susceptibility continues to matter, no matter the model specification or measures used. In other words, we failed to eliminate misinformation susceptibility as a substantial explainer of variance in vaccine skepticism. In addition, contrary to our expectations, we find no evidence that trust *moderates* the relationship between misinformation susceptibility and vaccine skepticism. Individuals who are susceptible to misinformation tend to be skeptical of vaccines, regardless of their trust level. This finding challenges the notion that trust could function as a “buffer” against misinformation^5^, and suggests instead that trust and misinformation are two distinct predictors for vaccine skepticism (though the two measures are correlated). This suggests that even those with high trust in official institutions can still fall prey to misinformation.

Though our study provides insights into the relationship between institutional (dis)trust, misinformation, and vaccine skepticism, we do note several limitations. First, the design of our study prevents us from making causal claims. Second, we were unable to include a longitudinal analysis. While our study did not find a moderating role of trust, future research may explore whether increased susceptibility to misinformation eventually erodes trust, thereby creating a cyclical relationship. Some research has shown that the impacts of mis- and disinformation on attitudes and beliefs take place over longer time spans (i.e., several years^44^), and it may well be the case that prolonged exposure to misinformation reinforces the erosion of trust. Third, while we collected a high-quality sample, we only did so in a single country and we caution against a universal interpretation of our findings. Fourth while we hypothesized trust as a protective factor against misinformation, emerging research suggests that trust may also increase susceptibility to misleading claims when such messages appear to come from trusted sources^45^. Future studies should examine this dual role of trust, especially in relation to source credibility and critical evaluation processes. Fifth, although our outcome variables and one of the trust measures are domain-specific (e.g., vaccination), the MIST captures *general* susceptibility to misinformation. While this scale is designed to assess individuals’ overarching skill to discern true from false information, which is known to relate to susceptibility in specific domains as well^36^. That said, future work could benefit from incorporating health-specific misinformation metrics as one could ideally compare domain-specific trust with domain-specific misinformation susceptibility (e.g., health-related misinformation). To our knowledge, no validated health-specific version of the MIST currently exists, which could be a valuable direction for future research. Moreover, future research could also benefit incorporating Signal Detection Theory (SDT) metrics in studies within this area of research^46,47^.

## Materials and methods

We used data collected in the context of the SocioVax research program carried out by the Dutch National Institute for Public Health and the Environment. All methods were carried out in accordance with internal guidelines. Data were collected through the LISS (Longitudinal Internet Studies for the Social Sciences) panel administered by Centerdata (Tilburg University, The Netherlands). The panel is based on a true probability sample of Dutch households, drawn from the population register by Statistics Netherlands. All respondents in this study provided informed consent prior to their participation. All LISS research projects and questionnaires that collect (special) personal data also require approval from the Centerdata Data Protection Officer and Information Security Officer, following the internal ‘Privacy, Information Security, and Data Management checklist’. Additionally, each LISS panel questionnaire undergoes an internal review, focusing on both language use and content, before being fielded to the panel. The Cambridge Psychology Research Ethics Committee has given ethical approval to the project of the Misinformation Susceptibility Test (Application no. PRE.2019.108). LISS panel members complete online questionnaires every month with a total duration of 60 min on average and receive a monetary incentive (€15 per hour) for each completed questionnaire. In total, 1,356 respondents filled in the questionnaire, among which people with children in the age between 0 and 14 (*n* = 678) and young adults without children (potential future parents, *n* = 678). The *n* was lower than expected in our preregistration, mainly because of a lower-than-anticipated response rate, which could be attributed to several factor such as (lack of) motivation of vaccine hesitant individuals or survey fatigue. Sample descriptive statistics are shown in Appendix 1. We used R studio (4.4.0) for regression, RF and moderation analyses. This study was preregistered at: https://aspredicted.org/ei25q.pdf. All scripts and relevant supplementary information are available at our OSF page. Data will be made public after publication due to confidentiality.

### Measures

We included high-quality, validated measures of misinformation susceptibility, institutional trust, and vaccine skepticism. See Appendix 2 for details on how variables were recorded in our dataset, as well as the full item wordings and the results of our exploratory and confirmatory factor analyses.

- For misinformation susceptibility, we used a translated version of the Misinformation Susceptibility Test or MIST^36^, a psychometrically validated instrument that measures an individuals’ general susceptibility to misinformation. While misinformation spreads across various platforms, the current study focuses on individuals’ general ability to discern true from false information. The MIST is designed to assess a broader, platform-independent susceptibility to misinformation. In a random order, respondents were shown six true and six false headlines and asked to indicate whether it is true or false, using a binary scale. Susceptibility to misinformation is assessed trough three different ability scores: real news detection ability, fake news detection ability and veracity discernment. We use “veracity discernment” (calculated as the sum of all correct responses on both the true and false headlines) as our main measure of interest. The Dutch-language version consists of 12 items, somewhat different from the 8-item, 16-item and 20-item English-language MIST versions developed by Maertens and colleagues^36^. We included 12 rather than 8 or 16 items due to the fact that we could only include a limited number of items in the LISS panel, and opted to maximize the number of items under this limitation. In translating the headlines, we adhered as closely as possible to the original wording, and used headlines that were most relevant within the Dutch context. The Dutch MIST has excellent fit measures (CFI = 0.945, TLI = 0.932, RMSEA = 0.041, SRMR = 0.071) and it can therefore be used as a reliable measure of misinformation susceptibility in the Dutch context. See Supplementary Information, Sect. 2.1.5 for more details.
- Institutional trust is a measure of how people perceive the quality of and their association with various institutions, often used in democratic countries^48,49^. Trust is often defined as “*a person’s belief that another person or institution will act consistently with their expectations of positive behavior*”^50^. It remains debated how trust should be measured exactly. Some scholars have argued that institutional trust could be assessed as an one-dimensional construct^30^, while others argue against ‘generalization’ when measuring trust^31^. This debate relates to discussions around to what extent government is perceived by citizens as one amorphous entity, or whether they differentiate between its different parts. We followed OECD guidelines to measure trust in institutions^50^. Respondents were asked on a scale from 1 to 10 to what extent they trust various institutions (1 is no trust at all and 10 is complete trust). We selected institutions that could plausibly influence vaccine attitudes or behaviors (institutions such as the police and the justice system were therefore excluded because they are not directly or indirectly related to vaccinations). Based on this selection, we conducted a series of Exploratory and Confirmatory Factor Analyses (see Appendix 3 for details). (In our preregistration, we mentioned to measure confidence of respondents in the following institutions (government, health care providers, science, media, politicians and political parties). We deviated from our registration, because we found insufficient fit measures for our initial scale and therefore deemed it more prudent to conduct an EFA and CFA.) We found that the institutional trust measures could be substantially improved by excluding some institutions from the scales (such as Health Care and Science), which may be explained because trust levels in these institutions are usually higher than others, e.g., parliament. In sum, we concluded that institutional trust can be broken down into two distinct scales, **A** and **B**. Institutional trust **A** includes the following institutions: parliament, politicians, political parties and the media. Institutional trust **B** primarily includes health-related institutions such as the National Institute of Public Health (coordinator of the National Vaccination Program), Municipal Health Services (executing organization) and GPs. However, these general trust measures do not capture issue-specific trust, particularly trust in how the government handles vaccination, a central concern in our study. Therefore, we included a scale (*specific trust)* targeting perceptions of the government with respect to vaccination policy. This is consistent with theoretical distinctions made in the literature between diffuse/system-level trust and specific/issue-based trust^51^. Including both types of trust allows us to examine whether general institutional trust and specific policy trust function differently in predicting vaccine skepticism. This scale addressed subdimensions of trust, namely perceptions of goverments’ capability, honesty, and intentions with respect to vaccines^52^ (See Supplementary Information 2.1.1–2.1.4) for more details). Respondents were asked the following question, on a Likert-scale from 1–5 (1 is fully disagree, 5 is fully agree): *The Dutch government determines the policy for vaccinations. What do you think about our government when it comes to vaccinations? Indicate to what extent you (dis)agree with the statements.*
  - “*The government has sufficient knowledge and skills regarding vaccinations*”,
  - “*The government communicates honestly about vaccinations*”,
  - “*The government acts in the interest of citizens when it comes to vaccination”.*
- We use three dependent variables for vaccine skepticism: Vaccine concern (i.e., general worry about vaccine safety), vaccine hesitancy (i.e., indecisiveness about whether to vaccinate one’s child), and vaccine refusal (i.e., actual non-uptake of vaccinations). Full definitions and operationalizations of these outcomes are explained in paragraph 2.1–2.3 in the Supplementary Material. For vaccine concern, we used items of the validated CABI-V questionnaire^53^ and constructed a variable by asking respondents to what extent they agree with three questions such as “*I worry that the ingredients in vaccinations are unsafe for children”* (See Appendix 3 for details for this measure). For vaccine hesitancy, we used a definition that has been proposed in a recent systematic literature review, which concluded that the term is best defined as “*a state of indecisiveness regarding a vaccination decision*”, and that hesitancy should be measured within the context of vaccinations^54^. Hence, we only use hesitancy as a measure for respondents that indicated having children. We used two measures of vaccine hesitancy, one for the main analysis and one to serve as a robustness check (see Appendix 2 for details). For vaccine refusal, we asked respondents whether their (youngest) child had received no vaccinations, some vaccinations or all vaccinations for which their child has been invited so far.

### Deviations from the preregistration

We preregistered to conduct linear regression to test H1. In the main article, we opted for non-parametric permutation tests as we deemed this method more prudent. For transparency, we report the results of the preregistered analyses in Appendix 3.1. We note that the results of both methods are highly comparable: small to moderate correlations are observed between the trust measures and misinformation susceptibility, also after controlling for confounders. In addition, we conducted Random Forest (RF) analyses to assess predictor importance for vaccine concern and hesitancy, which is a better method to assess predictor importance. In our preregistration, we stated that we would fit various linear regression models to assess predictors of vaccine concern and hesitancy. The results of the preregistered analyses are reported in Appendix 3.2. We note again that the outcomes are similar to the outcomes of the RF model: specific trust and institutional trust B are the strongest predictors for vaccine concern, and veracity discernment remains a significant predictor throughout all models, controlling for confounding factors.

## Supplementary Information

Below is the link to the electronic supplementary material.

Supplementary Material 1

## Data Availability

All scripts and relevant supplementary information are available at our OSF page. https://osf.io/4ua3t/?view_only=706518642af04abd8a018a00410fa406. Data will be made public after publication due to confidentiality.
