# Supplementary material for: Trust in institutions and misinformation susceptibility both independently explain vaccine skepticism
Source: Sci Rep. 2025 Oct 28;15:37655. doi: 10.1038/s41598-025-21452-1 (PMC12569172; doi:10.1038/s41598-025-21452-1)
Supplement: Supplementary file 1 — Supplementary Material 1 [file 41598_2025_21452_MOESM1_ESM.docx]

**Trust in institutions and misinformation susceptibility both independently explain vaccine skepticism**

**Supplementary information**

**This file includes:**

Appendix 1: descriptives

Appendix 2: explanation of variables and fit measures

Appendix 3: supplementary information for results presented in main article

**Appendix 1. Descriptives**

The questionnaire was presented to 2,016 LISS-panel members. 1356 members responded, of which 1,269 respondents completed the questionnaire in full (response rate 62.9%). For this measurement, respondents from target group 1 ((Young) adults (18 to 40 years) without children) and target group 2 (Parents of children (up to 14 years)) were invited.

*Table S1.* Sample descriptives.

| **Variable** | **Overall** | **Group** | |
| --- | --- | --- | --- |
|  | N = 1,356 | **1^a^** | **2^b^** |
|  |  | N = 678 | N = 678 |
| **Age Category** |  |  |  |
| 15 - 24 years | 213 (16%) | 209 (31%) | 4 (0.6%) |
| 25 - 34 years | 469 (35%) | 336 (50%) | 133 (20%) |
| 35 - 44 years | 481 (35%) | 133 (20%) | 348 (51%) |
| 45 - 54 years | 173 (13%) | 0 (0%) | 173 (26%) |
| 55 - 64 years | 17 (1.3%) | 0 (0%) | 17 (2.5%) |
| 65 years and older | 3 (0.2%) | 0 (0%) | 3 (0.4%) |
| **Gender** |  |  |  |
| Male | 584 (43%) | 285 (42%) | 299 (44%) |
| Female | 766 (56%) | 387 (57%) | 379 (56%) |
| Other | 6 (0.4%) | 6 (0.9%) | 0 (0%) |
| **Education Category** |  |  |  |
| Primary education | 53 (3.9%) | 44 (6.5%) | 9 (1.3%) |
| vmbo | 77 (5.7%) | 37 (5.5%) | 40 (5.9%) |
| havo/vwo | 157 (12%) | 129 (19%) | 28 (4.1%) |
| mbo | 349 (26%) | 120 (18%) | 229 (34%) |
| hbo | 382 (28%) | 155 (23%) | 227 (34%) |
| wo | 331 (25%) | 188 (28%) | 143 (21%) |
| **Income Category (net montly €)** |  |  |  |
| No income | 128 (9.5%) | 87 (13%) | 41 (6.0%) |
| 1 to 1500 | 257 (19%) | 165 (25%) | 92 (14%) |
| 1501 to 2500 | 353 (26%) | 155 (23%) | 198 (29%) |
| 2501 to 3500 | 384 (29%) | 168 (25%) | 216 (32%) |
| 3501 to 4500 | 110 (8.2%) | 42 (6.3%) | 68 (10%) |
| 4501 and higher | 115 (8.5%) | 52 (7.8%) | 63 (9.3%) |
| **Origin** |  |  |  |
| Dutch origin | 963 (71%) | 460 (68%) | 503 (74%) |
| Migrant or child of migrant(s) | 388 (29%) | 214 (32%) | 174 (26%) |

a Young adults (18 to 40 years of age) without children

b Parents of children (up to 14 years of age)

**Appendix 2 explanation of variables and fit measures**

1. **Independent Variables**
   1. **Institutional trust (EFA)**

We started by conducting an Exploratory Factor Analysis of the institutional trust items we possessed, making a selection of institutions that could plausibly affect vaccination attitudes. Respondents were asked the following questions: *“Can you indicate, on a scale from 0 to 10, how much confidence you personally have in each of the following institutions”*, following OECD-guidelines (see references):

- V52a The government
- V52b Municipal health services
- V52c The National Institute of Public Health
- V52d GP (general practitioner)
- V52f Pharmaceutical companies
- cv24p014 The Dutch Parliament
- cv24p017 Politicians
- cv24p018 Political parties
- cv24p021 The media
- cv24p025 Science

Parallel analysis showed that the trust items potentially consist of 2 to 3 constructs.


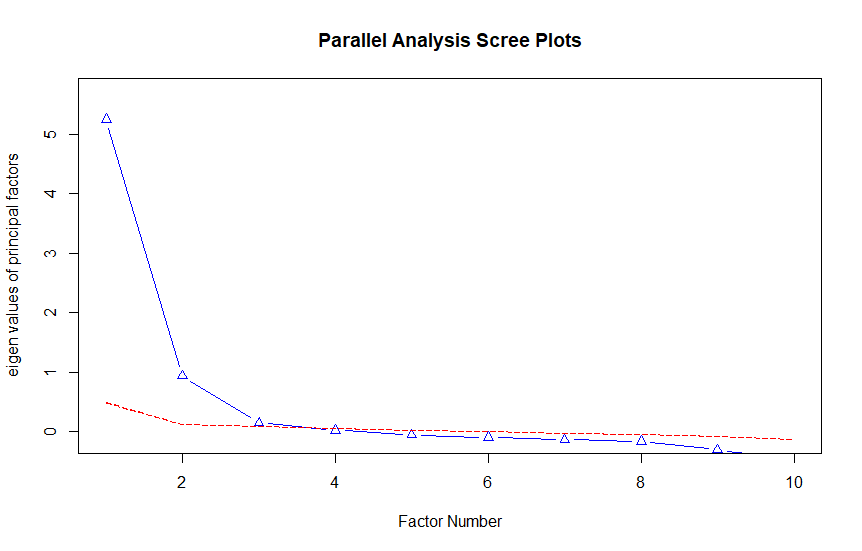


*Fig. S1*. Parallel analysis (EFA) of trust items.

Results of EFA:

*Table S2.* Factor loadings, variance metrics and factor correlations of EFA for institutional trust items.

| **Variable** | **Factor 1** | **Factor 2** | **Factor 3** |
| --- | --- | --- | --- |
| V52a | 0.445 |  | 0.543 |
| V52b | 0.966 |  |  |
| V52c | 0.876 |  |  |
| V52d | 0.790 |  |  |
| V52f | 0.504 |  |  |
| cv24p014 |  | 0.780 |  |
| cv24p017 |  | 1.026 |  |
| cv24p018 |  | 1.017 |  |
| cv24p021 |  | 0.535 |  |
| cv24p025 | 0.525 |  |  |
| **Metric** |  |  |  |
| SS loadings | 3.106 | 3.069 | 0.431 |
| Proportion Var | 0.311 | 0.307 | 0.043 |
| Cumulative Var | 0.311 | 0.618 | 0.661 |
| **Factor Correlations** |  |  |  |
| Factor 1 | 1.000 | 0.642 | -0.474 |
| Factor 2 | 0.642 | 1.000 | -0.475 |
| Factor 3 | -0.474 | -0.475 | 1.000 |

- 1. **CFA: Institutional Trust A**

EFA suggested that items cv24p014, cv24p017, cv24p018, cv24p021 load onto a single factor.

*Can you indicate, on a scale from 0 to 10, how much confidence you personally have in each of the following institutions:*

- V52b The Dutch Parliament
- V52c Politicians
- V52d Political parties
- V52f The media

CFA was conducted for the scale (Institutional trust A) to check fit measures. We conclude this scale shows good fit measures:

*Table S3.* Fit measures for Institutional Trust A variable

| **Chisq** | **df** | ***p*-value** | **CFI** | **TLI** | **RMSEA** | **SRMR** | **Cronbach’s Alpha** |
| --- | --- | --- | --- | --- | --- | --- | --- |
| 2.815 | 2.000 | 0.245 | 1.000 | 0.999 | 0.020 | 0.006 | 0.90 |

We also report the inter-correlations for this variable:

*Table S4.* Correlation matrix of Institutional Trust A variable

|  | **V52b** | **V52c** | **V52d** | **V52f** |
| --- | --- | --- | --- | --- |
| **V52b** | 1 | 0.866 | 0.653 | 0.615 |
| **V52c** | 0.866 | 1 | 0.604 | 0.630 |
| **V52d** | 0.653 | 0.604 | 1 | 0.470 |
| **V52f** | 0.615 | 0.631 | 0.470 | 1 |

- 1. **CFA: Institutional Trust B**

EFA suggested that items v52a, v52b, v52c, v52d, v52f, and cv24p025 could potentially construct factor, although v52a loads on a third factor too. CFA however showed insufficient fit measures or this scale (RMSEA = 1.02). Item cv24p021 showed the lowest factor loading (0.535). Hence, we improved the scale by removing v52a and cv24p025. The improved scale of the Institutional trust B is constructed by the following questions:

*“Can you indicate, on a scale from 0 to 10, how much confidence you personally have in each of the following institutions”.*

- cv24p014 Municipal Health Services
- cv24p017 The National Institute of Public Health
- cv24p018 General Practitioner
- cv24p021 Pharmaceutical companies

CFA was conducted for the improved scale to check fit measures. We conclude this scale is sufficient:

*Table S5.* Fit measures for Institutional Trust B variable.

| **Chisq** | **df** | ***p*-value** | **CFI** | **TLI** | **RMSEA** | **SRMR** | **Cronbach’s Alpha** |
| --- | --- | --- | --- | --- | --- | --- | --- |
| 18.183 | 2.000 | 0.000 | 0.994 | 0.981 | 0.089 | 0.013 | 0.87 |

*Note:* the chi-square test is significant, indicating a mismatch between the observed and model-implied covariance matrices. However, this test is very sensitive to large sample sizes: other fit indices are considered to be considered for a more balanced interpretation.

We also report the inter-correlations for this variable:

*Table S6*. Correlation matrix institutional trust B

|  | **cv24p014** | **cv24p017** | **cv24p018** | **cv24p21** |
| --- | --- | --- | --- | --- |
| **cv24p014** | 1 | 0.715 | .677 | 0.486 |
| **cv24p017** | 0.715 | 1 | 0.855 | 0.611 |
| **cv24p018** | 0.677 | 0.855 | 1 | 0.581 |
| **cv24p021** | 0.486 | 0.612 | 0.58 | 1 |

- 1. **Specific trust**

As mentioned in the main article, it remains debated how to measure trust in what circumstances. Some scholars advocate for a unidimensional construct of institutional trust, while others emphasize the need to disaggregate trust into more domain-specific or context-specific components, cautioning against overly broad generalizations (e.g., Levi & Stoker, 2000; Newton & Zmerli, 2011). In our study, we sought to balance these perspectives by including both generalized trust in institutions and a specific trust measure related to vaccination policy. For the general trust indicators (Institutional Trust A and B), we followed OECD guidelines for assessing institutional trust, focusing on institutions plausibly relevant to vaccine policy and implementation. Through exploratory factor analysis, we found that these institutions grouped empirically into two distinct clusters: Trust A: primarily political institutions (e.g., parliament, national government) Trust B: health-related institutions (e.g., public health services, general practitioners). We treated these as two conceptually and empirically distinct subdimensions, reflecting the idea that individuals may differentiate between political actors and healthcare providers in their evaluations.

However, these general trust measures do not capture issue-specific trust, particularly trust in how the government handles vaccination—a central concern in our study. Therefore, we included a specific trust item targeting perceptions of the government with respect to vaccination policy. This is consistent with theoretical distinctions made in the literature between diffuse/system-level trust and specific/issue-based trust (e.g., Siegrist, 2021). Including both types of trust allows us to examine whether general institutional trust and specific policy trust function differently in predicting vaccine skepticism. For specific trust, respondents were asked to answer the following questions, on a Likert scale from 1-5 (1 is fully disagree and 5 is fully agree).

- v49a The government has sufficient knowledge and skills regarding vaccinations
- v49b The government communicates honestly about vaccinations.
- v49c The government acts in the interest of citizens when it comes to vaccinations

*Table S7.* Correlation matrix for specific trust variable

|  | **V49a** | **V49b** | **V49c** |
| --- | --- | --- | --- |
| **V49a** | 1 | 0.77 | 0.769 |
| **V49b** | 0.774 | 1 | 0.847 |
| **V49c** | 0.769 | 0.847 | 1 |

We conducted a One Factor CFA for the scale. Fit measures such as CFI and TLI were not calculated because this scale consists of only 3 items. The internal consistency (Cronbach’s Alpha) of the specific trust scale is 0.92, suggesting that the three items (V49a, V49b, and V49c) are highly correlated and reliably measure the same underlying construct (specific trust). The one-factor CFA results suggest that the scale is reliable and valid for measuring the underlying construct (specific trust). The high factor loadings and excellent internal consistency indicate that the three items effectively capture a unidimensional construct.

*Table S8.* CFA for Specific Trust variable

| **Factor loadings** | **Estimate** | **Std.Err** | **Z-value** | **P(>\|z\|)** |
| --- | --- | --- | --- | --- |
| V49a | 1.000 |  |  |  |
| V49b | 1.141 | 0.028 | 41.049 | 0.000 |
| V49c | 1.129 | 0.028 | 40.775 | 0.000 |
| **Variances:** |  |  |  |  |
| V49a | 0.289 | 0.015 | 19.839 | 0.000 |
| V49b | 0.152 | 0.013 | 12.015 | 0.000 |
| V49c | 0.164 | 0.013 | 12.863 | 0.000 |

*EFA of all used trust items*

In order to test the assumption whether the generic trust measures and specific trust capture different factors, we conducted an additional EFA:

*Table S9.* Extra EFA: Factor loadings, variance metrics and factor correlations for different trust measures

| **Variable** | **Inst. Trust A** | **Inst. trust B** | **Specific Trust** |
| --- | --- | --- | --- |
| V52b |  | 1.019 |  |
| V52c |  | 0.830 |  |
| V52d |  | 0.671 |  |
| V52f |  | 0.532 |  |
| cv24p014 | 0.786 |  |  |
| cv24p017 | 1.008 |  |  |
| cv24p018 | 0.999 |  |  |
| cv24p021 | 0.534 |  |  |
| V49a |  |  | 0.838 |
| V49b |  |  | 0.880 |
| V49c |  |  | 0.892 |
| **Metric** |  |  |  |
| SS loadings | 2.950 | 2.488 | 2.298 |
| Proportion Var | 0.268 | 0.226 | 0.209 |
| Cumulative Var | 0.268 | 0.494 | 0.703 |
| **Factor Correlations** |  |  |  |
| Inst. Trust A | 1 | 0.605 | 0.776 |
| Inst. Trust B | 0.605 | 1 | 0.512 |
| Specific Trust | 0.776 | 0.512 | 1 |

*Note. The results show that generic and specific trust items clearly load on different factors, with no-cross-loadings. We interpret this as support for our claim that these variables are distinct factors, and that they are not measuring the same underlying dimension, even though they are moderately correlated.*

- 1. **Misinformation Susceptibility**

We used a translated version of the Misinformation Susceptibility Test (MIST) (Maertens et al., 2023), a psychometrically validated instrument that measures an individuals’ general susceptibility to misinformation. Respondents were shown 6 true and 6 false headlines and asked to judge whether it is true or false, using a binary scale. In this test, susceptibility to misinformation is assessed trough three different ability scores: *veracity discernment* *(V)* an individual’s overall accuracy in discerning true and false headlines, *real news detection* ability *(R)* to identify real headlines, and fake news detection skill (*F*) skill to identify false headlines.


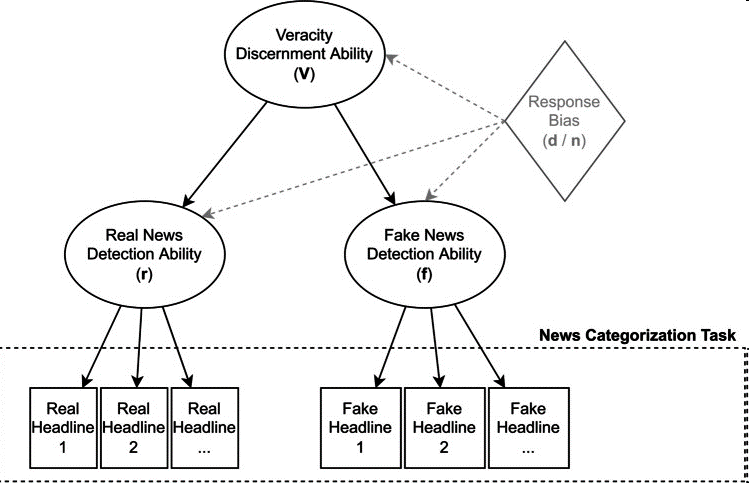


*Fig. S2.* Veracity Discernment ability. From Maertens et al. (2024).

The following headlines were asked in random order:

*Fake headlines (if = item false)*

If1 Government Officials Have Manipulated Stock Prices to Hide Scandals

If2 New Study: Left-Wingers Are More Likely to Lie to Get a Higher Salary

If3 New Study: Clear Relationship Between Eye Color and Intelligence

If4 The Government Is Knowingly Spreading Disease Through the Airwaves and Food Supply

If5 A Small Group of People Control the World Economy by Manipulating the Price of Gold and Oil

If6 Climate Scientists' Work Is 'Unreliable', a 'Deceptive Method of Communication'

*Read headlines: (ir = item right)*

Ir7 Attitudes towards EU Are Largely Positive, Both Within Europe and Outside It

Ir8 Hyatt Will Remove Small Bottles from Hotel Bathrooms by 2021

Ir9 Republicans Divided in Views of Trump’s Conduct, Democrats Are Broadly Critical

Ir10 United Nations Gets Mostly Positive Marks from People Around the World

Ir11 Taiwan Seeks to Join Fight Against Global Warming

ir12 Morocco’s King Appoints Committee Chief to Fight Poverty and Inequality

Veracity Discernment scores were calculated by summing up the correctly identified fake and real headlines, resulting in a score between 0-12. Both exploratory (EFA) and confirmatory factor analysis (CFA) were conducted:


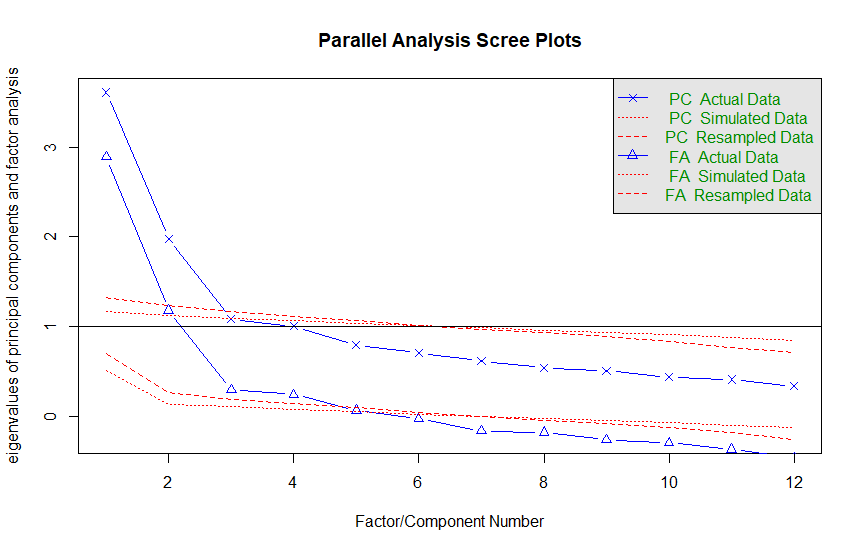


*Fig. S3*. Parallel analysis for Misinformation Susceptibility.

The eigenvalues suggested 3 or 4 factors. However, 4 factors yield uninterpretable factors (i.e., factors with less than 3 items, cross-loadings, etc.). Confirmatory factor analysis (CFA) with Lavaan package in accordance with the theoretical second order model (see Fig. S2 above) from Maertens et al. (2024), showed the following results:

*Table S10.* Model fit indices of the CFA for Veracity Discernment

| **Fit Index** | **Value** | **Scaled Value** |
| --- | --- | --- |
| Chi-square Test | 166.30 | 196.85 |
| Degrees of freedom (df) | 53 | 53 |
| p-value (Chi-square) | < 0.001 | < 0.001 |
| Comparative Fit Index (CFI) | 0.943 | 0.909 |
| Tucker-Lewis Index (TLI) | 0.929 | 0.887 |
| Robust CFI | — | 0.838 |
| Robust TLI | — | 0.798 |
| RMSEA | 0.042 | 0.048 |
| 90% CI for RMSEA | [0.035, 0.050] | [0.041, 0.055] |
| SRMR | 0.073 | 0.073 |

*Table S11.* Residual variances of the Misinformation Susceptibility Test

| **Item** | **Estimate** | **Std. Error** | **z-value** | **p-value** | **Standardized** |
| --- | --- | --- | --- | --- | --- |
| fF (Fake news detection factor) |  |  |  |  |  |
| if1 | 1.000 |  |  |  | 0.555 |
| if2 | 1.074 | 0.111 | 9.682 | < 0.001 | 0.596 |
| if3 | 0.760 | 0.100 | 7.622 | < 0.001 | 0.422 |
| if4 | 1.459 | 0.130 | 11.210 | < 0.001 | 0.809 |
| if5 | 1.128 | 0.107 | 10.568 | < 0.001 | 0.626 |
| if6 | 1.088 | 0.108 | 10.105 | < 0.001 | 0.604 |
| fR (Real news detection factor) |  |  |  |  |  |
| ir7 | 1.000 |  |  |  | 0.668 |
| ir8 | 0.776 | 0.086 | 9.063 | < 0.001 | 0.518 |
| ir9 | 0.892 | 0.084 | 10.579 | < 0.001 | 0.596 |
| ir10 | 0.838 | 0.079 | 10.611 | < 0.001 | 0.560 |
| ir11 | 0.862 | 0.083 | 10.421 | < 0.001 | 0.575 |
| ir12 | 0.861 | 0.083 | 10.370 | < 0.001 | 0.575 |
| fV (Veracity Discernment factor fV =~ fF + fV) |  |  |  |  |  |
| ~fF | 1.000 |  |  |  | 0.709 |
| ~fR | 1.000 |  |  |  | 0.589 |

*Table S12.* Standardized Factor Loadings for the Misinformation Susceptibility Test

| **Item** | **Estimate** | **Std. Error** | **z-value** | **p-value** | **Standardized** |
| --- | --- | --- | --- | --- | --- |
| if1 | 0.692 |  |  |  | 0.692 |
| if2 | 0.645 |  |  |  | 0.645 |
| if3 | 0.822 |  |  |  | 0.822 |
| if4 | 0.345 |  |  |  | 0.345 |
| if5 | 0.609 |  |  |  | 0.609 |
| if6 | 0.636 |  |  |  | 0.636 |
| ir7 | 0.554 |  |  |  | 0.554 |
| ir8 | 0.731 |  |  |  | 0.731 |
| ir9 | 0.645 |  |  |  | 0.645 |
| ir10 | 0.687 |  |  |  | 0.687 |
| ir11 | 0.669 |  |  |  | 0.669 |
| ir12 | 0.669 |  |  |  | 0.669 |
| fF(Fake news detection factor) | 0.153 | 0.042 | 3.647 | 0.000 | 0.497 |
| fR(Real news detection factor | 0.291 | 0.051 | 5.717 | 0.000 | 0.653 |
| fV(Veracity Discernment factor) | 0.155 | 0.024 | 6.402 | 0.000 | 1.000 |

*Table S13*. Internal consistency of the Misinformation Susceptibility Test

| **Factor** | **Cronbach’s Alpha** |
| --- | --- |
| Fake news detection ability | 0.77 |
| Real news detection ability | 0.75 |
| Veracity Discernment ability | 0.78 |

1. **Dependent variables**

We differentiate between (1) vaccine concern (2) vaccine hesitancy and (3) vaccine refusal. We follow the definition that has been proposed in a recent systematic literature review, which concludes that vaccine hesitancy could best be described as “*a state of indecisiveness regarding a vaccination decision” (Bussink-Voorend et al., 2022*). Importantly, this view distinguishes between vaccination hesitancy as a psychological state and vaccine refusal as a behavior. Several authors have argued that these concepts are related but should be differentiated (Bedford et al., 2018; Díaz Crescitelli et al., 2020).

One reason is that studying only actual behavior fails to properly discriminate between hesitant and non-hesitant people. Some individuals may choose to vaccinate their children with or without hesitation, and similarly some may reject vaccinations with or without hesitation. Similarly, while certain types of vaccination behaviors (refusal, for instance), could indeed be manifestations of vaccine hesitancy, this is does not mean that every vaccine-refusing individual is also vaccine-hesitant. On the other hand, studying actual behavior is also important because vaccine uptake in practice matters for herd immunity. Another reason to distinguish between hesitancy as a psychological state and refusal as a behavior, is that we know there is a difference between intentions of behavior and actual behavior, often referred to as the ‘intention-behavior’ gap (Conner & Norman, 2022). In the context of vaccinations, while some individuals may have positive attitudes towards or even intend to vaccinate their children, their actual behaviors in practice might not correspond with their intentions.

- 1. **Vaccine Concern**

We used the validated CABI-V questionnaire to measure vaccine concern (Shoup, 2015) All respondents were asked on a Likert-scale from 1-5 to what extent they agree with the following statements:

- V37 I worry that the ingredients in vaccinations are unsafe for children
- V38 I worry about children feeling sick shortly after getting a vaccination
- V39 I worry that vaccinations have serious side effects for children
- V40 I worry that children's immune systems are weakened by vaccinations

Note: item v38 *(“I worry about children feeling sick shortly after getting a vaccination”)* is not part of the original CABI-V questionnaire. We included this item to add more nuance to the measure, as it’s possible that individuals may not be concerned about serious side effects of vaccines (v39), but could still be worried about minor side effects that occur shortly after vaccination.

*Table S14.* Model fit measures of Vaccine Concern:

| **Chisq** | **df** | **p-value** | **CFI** | **TLI** | **RMSEA** | **SRMR** | **Cronbach’s Alpha** |
| --- | --- | --- | --- | --- | --- | --- | --- |
| 12.528 | 2.000 | 0.002 | 0.997 | 0.990 | 0.066 | 0.010 | 0.90 |

*Table S15* Correlation matrix of Vaccine Concern

|  | **V37** | **V38** | **V39** | **V40** |
| --- | --- | --- | --- | --- |
| **V37** | 1 | 0.577 | 0.815 | 0.758 |
| **V38** | 0.577 | 1 | 0.637 | 0.569 |
| **V39** | 0.815 | 0.637 | 1 | 0.767 |
| **V40** | 0.758 | 0.569 | 0.767 | 1 |

- 1. **Vaccine Hesitancy**

Vaccine Hesitancy is a construct that should only be measured in the context of vaccinations Bussink-Voorend et al., 2024). Hence, we only used this measure for the people who indicated to have children. We used two measures for vaccine hesitancy, one for the main analyses and one as a robustness check. Moreover, there is no unequivocal translation for vaccine hesitancy to Dutch – which is an issue for various languages. To measure vaccine hesitancy, we used a definition and operationalization according to Bussink-Voorend et al. (2024). We used an additional measure (vaccine hesitancy B) as a robustness check).

**Vaccine Hesitancy A**

*Please think about the next vaccination that your youngest child will be offered. To what extent do you have doubts about whether or not to have your youngest child vaccinated? Choose the number that suits you best.*

*No doubt 1 – 2 – 3 – 4 – 5 – 6 – 7 – 8 – 9 – 10 A lot of doubt*

**Vaccine Hesitancy B**

Respondents were asked the following question on a Likert scale 1-5:

*Think about the next vaccination that your youngest child will be offered. When it comes to vaccinating my youngest child*

- V8a I know what the best choice is for my child.
- V8b I feel confident about the choice.
- V8c I can easily make the decision.

*Table S16.* Correlation of Matrix Vaccine hesitancy variable

|  | **V8a** | **V8b** | **V8c** |
| --- | --- | --- | --- |
| **V8a** | 1 | 0.655 | 0.702 |
| **V8b** | 0.655 | 1 | 0.766 |
| **V8c** | 0.701 | 0.766 | 1 |

As shown in Table S15, the items correlate highly. The measure for internal consistency, Cronbach’s Alpha = 0.87, which indicates good internal consistency of this scale and that it measures the same construct. The scale is valid and reliable for measuring the intended construct.

*Table S17.* CFA for Vaccine Hesitancy B variable

| **Factor loadings** | **Estimate** | **Std.Err** | **Z-value** | **P(>\|z\|)** |
| --- | --- | --- | --- | --- |
| V8a | 1.000 |  |  |  |
| V8b | 1.139 | 0.052 | 21.989 | 0.000 |
| V8c | 1.137 | 0.051 | 22.513 | 0.000 |
| **Variances:** |  |  |  |  |
| V8a | 0.302 | 0.021 | 14.170 | 0.000 |
| V8b | 0.234 | 0.021 | 11.035 | 0.000 |
| V8c | 0.129 | 0.018 | 7.121 | 0.000 |

- 1. **Vaccine Refusal**

Respondents who indicated to have children were asked the following question:

*The National Vaccination Program in the Netherlands has several vaccinations for children and young people. Has your youngest child had none of these vaccinations, some vaccinations or all vaccinations? By this we mean the vaccinations for which your child has been invited so far.*

- *No vaccinations*
- *Some vaccinations*
- *All vaccinations*

**Appendix 3 supplementary information for results presented in main article**

**3.1. Regression analyses: predictors of institutional trust.** The following figure and three tables contain detailed information of the preregistered regression analysis. We fitted a series of linear regression models with three measures of institutional and specific trust as the dependent variables and veracity discernment (our reversed measure of misinformation susceptibility) as the key independent variable of interest (see Methods). Age, gender (recoded to male/female), income, education level, and origin (binary, Dutch vs migrants or children of migrants) were included as covariates. Veracity discernment is positively associated with institutional trust A, i.e., trust in political institutions (𝛽 = 0.226, *p* < .001). Similar effects are found for institutional trust B, i.e., trust in health institutions, 𝛽 = 0.334, *p* < .001, as well as for specific trust in government with respect to vaccinations, 𝛽 = 0.362, *p* < .001.


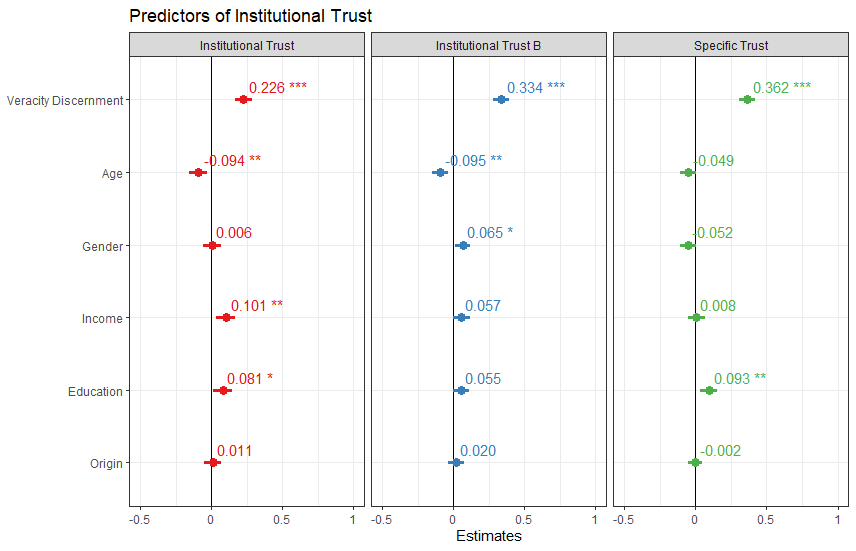


*Fig. S4.* Linear regression with veracity discernment, age, gender, income, education level, and ethnicity predicting institutional trust (n = 1356). Values shown are standardized estimates. * *p* < .05, ** *p* < .01, *** *p* < .001.

| *Table S18***.** Predictors of Institutional Trust A | | | | | |
| --- | --- | --- | --- | --- | --- |
|  | **Institutional Trust A** | | | | |
| *Predictors* | *Estimates* | *std. Beta* | *CI* | *standardized CI* | *p* |
| (intercept) | 27.605 | 0.0000 | 1.9567 – 3.5643 | -0.0592 – 0.0592 | **<0.001** |
| Veracity Discernment | 0.1916 | 0.2262 | 0.1389 – 0.2443 | 0.1640 – 0.2885 | **<0.001** |
| Age | -0.0203 | -0.0936 | -0.0342 – -0.0064 | -0.1577 – -0.0295 | **0.004** |
| Gender | 0.0247 | 0.0064 | -0.2122 – 0.2615 | -0.0548 – 0.0675 | 0.838 |
| Income | 0.0001 | 0.1010 | 0.0000 – 0.0001 | 0.0314 – 0.1707 | **0.005** |
| Education | 0.1239 | 0.0807 | 0.0202 – 0.2277 | 0.0132 – 0.1482 | **0.019** |
| Origin | 0.0005 | 0.0112 | -0.0021 – 0.0031 | -0.0481 – 0.0705 | 0.712 |
| Observations | 1006 | | | | |
| R^2^ / R^2^ adjusted | 0.090 / 0.084 | | | | |
| *Table S19.* Predictors of Institutional trust B | | | | | |
|  | **Institutional Trust B** | | | | |
| *Predictors* | *Estimates* | *std. Beta* | *CI* | *standardized CI* | *p* |
| (intercept) | 40.500 | 0.0000 | 3.3732 – 4.7268 | -0.0529 – 0.0529 | **<0.001** |
| Veracity Discernment | 0.2649 | 0.3345 | 0.2211 – 0.3088 | 0.2791 – 0.3898 | **<0.001** |
| Age | -0.0195 | -0.0950 | -0.0312 – -0.0078 | -0.1520 – -0.0381 | **0.001** |
| Gender | 0.2374 | 0.0653 | 0.0387 – 0.4362 | 0.0106 – 0.1200 | **0.019** |
| Income | 0.0000 | 0.0568 | -0.0000 – 0.0001 | -0.0035 – 0.1171 | 0.065 |
| Education | 0.0779 | 0.0546 | -0.0065 – 0.1623 | -0.0046 – 0.1139 | 0.071 |
| Origin | 0.0009 | 0.0197 | -0.0015 – 0.0033 | -0.0333 – 0.0726 | 0.466 |
| Observations | 1195 | | | | |
| R^2^ / R^2^ adjusted | 0.136 / 0.132 | | | | |
| *Table S20.* Predictors of Specific Trust | | | | | |
|  | **Specific Trust** | | | | |
| *Predictors* | *Estimates* | *std. Beta* | *CI* | *standardized CI* | *p* |
| (intercept) | 23.976 | -0.0000 | 2.0547 – 2.7405 | -0.0520 – 0.0520 | **<0.001** |
| Veracity Discernment | 0.1477 | 0.3622 | 0.1255 – 0.1700 | 0.3077 – 0.4166 | **<0.001** |
| Age | -0.0052 | -0.0493 | -0.0112 – 0.0007 | -0.1054 – 0.0067 | 0.084 |
| Gender | -0.0973 | -0.0520 | -0.1980 – 0.0034 | -0.1058 – 0.0018 | 0.058 |
| Income | 0.0000 | 0.0080 | -0.0000 – 0.0000 | -0.0513 – 0.0672 | 0.792 |
| Education | 0.0683 | 0.0931 | 0.0255 – 0.1111 | 0.0348 – 0.1513 | **0.002** |
| Origin | -0.0000 | -0.0018 | -0.0012 – 0.0012 | -0.0539 – 0.0503 | 0.945 |
| Observations | 1195 | | | | |
| R^2^ / R^2^ adjusted | 0.164 / 0.160 | | | | |

**3.2. Predictors of vaccine concern and hesitancy**

As mentioned in the main article, we conducted Random Forest (RF) analyses to assess predictor importance for Vaccine hesitancy. By doing so, we deviated from our preregistration, in which we stated that we would fit various linear regression models to assess predictors of vaccine concern and hesitancy. For transparency, we here report the results of the preregistered analyses. Note that the outcomes are similar than to the outcomes of the RF model: specific trust and institutional trust B are the strongest predictors for vaccine concern, and veracity discernment remains a significant predictor throughout all models, controlling for confounding factors.


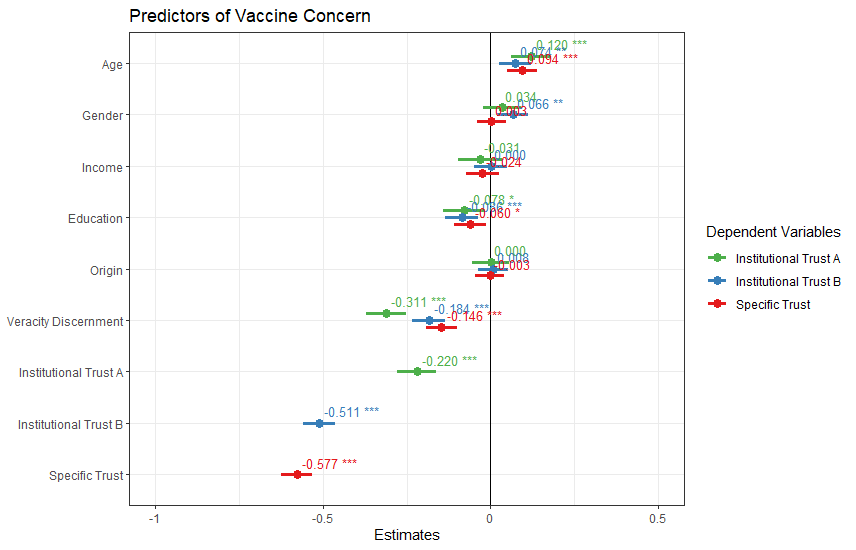


*Fig. S5.* Linear regression with age, gender, income, education level, origin, veracity discernment and various measures of trust predicting vaccine concern (*n* = 1356). Values shown are standardized estimates.* *p* < .05, ** *p* < .01, *** *p* < .001.

In addition to Vaccine Concern, we conducted the same statistical analyses (linear regression and random forest) for the vaccine hesitancy variable:
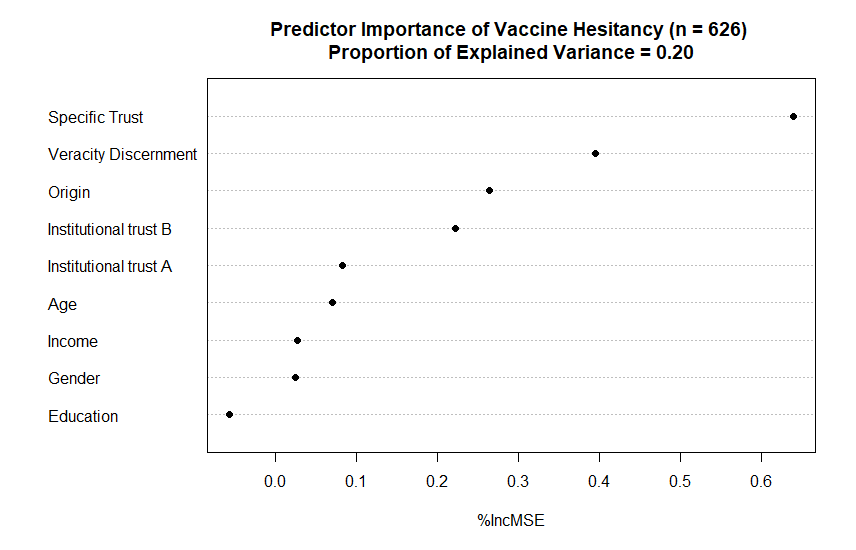


*Fig. S6.* Random Forest Analysis for Vaccine Hesitancy. The RF model explains 20% of the variance, which is lower than the RF model for Vaccine Concern. This could be due to the lower N of this RF model (we remind the author that vaccine hesitancy was only used as a construct for respondents with children, whereas vaccine concern was used for all respondents). The model achieved a Mean Absolute Error (MAE) of 0.57, which suggests that the model provides a reasonably accurate approximation of vaccine concern.


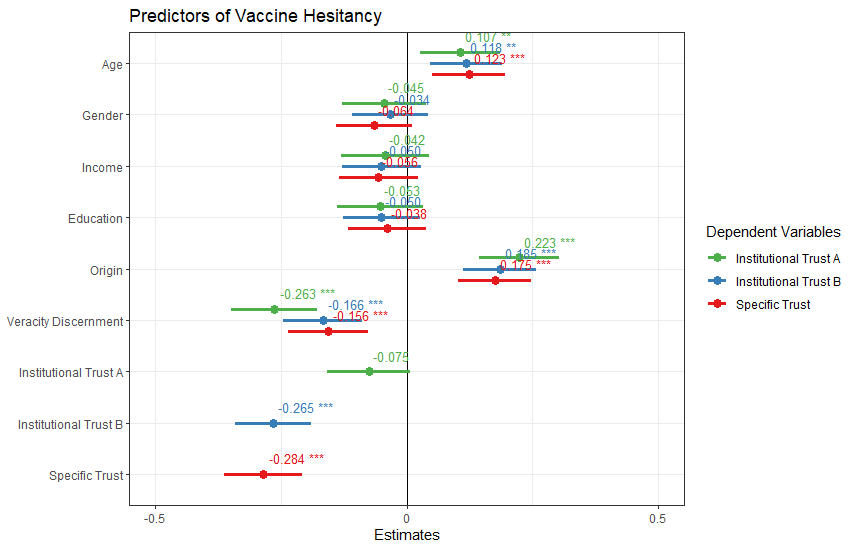


*Fig. S7.* Linear regression with age, gender, income, education level, origin, veracity discernment and various measures of trust predicting vaccine hesitancy (N = 626). Values shown are standardized estimates.* *p* < .05, ** *p* < .01, *** *p* < .001. Similar to the Random Forest analysis, specific trust is the strongest predictor in the models. Veracity discernment is a significant and robust predictor in all models.

**3.3. Moderation Analysis**

As mentioned in the main article, for the moderation analyses, we fitted a series of linear regression models predicting our two main dependent variables (vaccine concern and hesitancy), looking at the interaction terms between veracity discernment and our three measures of institutional trust, and the same demographic variables as covariates. We find no significant interaction effects in any of our models (all *p* values > .155) , as can be seen in tables below (see the bottom rows of each table for the interaction terms).

| *Table S21****.*** Interaction effects for Vaccine Concern with Institutional Trust B and Veracity Discernment as predictors | | | | | | |
| --- | --- | --- | --- | --- | --- | --- |
|  | **Vaccine Concern** | | | | | |
| *Predictors* | *Estimates* | *std. Beta* | *CI* | *standardized CI* | *p* | *std. p* |
| (intercept) | 3.834 | 0.005 | 3.273 – 4.394 | -0.040 – 0.051 | **<0.001** | 0.816 |
| Institutional Trust B | -0.223 | -0.493 | -0.300 – -0.145 | -0.542 – -0.443 | **<0.001** | **<0.001** |
| Veracity Discernment | -0.046 | -0.170 | -0.107 – 0.015 | -0.219 – -0.121 | 0.140 | **<0.001** |
| Age | 0.007 | 0.067 | 0.002 – 0.012 | 0.020 – 0.114 | **0.006** | **0.006** |
| Gender | 0.150 | 0.079 | 0.064 – 0.236 | 0.034 – 0.124 | **0.001** | **0.001** |
| Income | 0.000 | 0.001 | -0.000 – 0.000 | -0.049 – 0.050 | 0.977 | 0.977 |
| Education | -0.065 | -0.089 | -0.101 – -0.029 | -0.138 – -0.040 | **<0.001** | **<0.001** |
| Origin | 0.296 | 0.141 | 0.202 – 0.391 | 0.096 – 0.186 | **<0.001** | **<0.001** |
| **Institutional Trust B * Veracity Discernment** | -0.003 | -0.016 | -0.013 – 0.006 | -0.057 – 0.026 | 0.456 | 0.456 |
| Observations | 1187 | | | | | |
| R^2^ / R^2^ adjusted | 0.418 / 0.414 | | | | | |

| *Table S22.* Interaction effects for Vaccine Concern with Specific Trust and Veracity Discernment as predictors | | | | | | |
| --- | --- | --- | --- | --- | --- | --- |
|  | **Vaccine Concern** | | | | | |
| *Predictors* | *Estimates* | *std. Beta* | *CI* | *standardized CI* | *p* | *std. p* |
| (intercept) | 3.994 | 0.011 | 3.413 – 4.574 | -0.033 – 0.055 | **<0.001** | 0.625 |
| Specific Trust | -0.448 | -0.558 | -0.597 – -0.299 | -0.604 – -0.511 | **<0.001** | **<0.001** |
| Veracity Discernment | -0.011 | -0.137 | -0.073 – 0.050 | -0.185 – -0.090 | 0.720 | **<0.001** |
| Age | 0.009 | 0.086 | 0.004 – 0.014 | 0.041 – 0.131 | **<0.001** | **<0.001** |
| Gender | 0.035 | 0.019 | -0.047 – 0.118 | -0.025 – 0.062 | 0.400 | 0.400 |
| Income | -0.000 | -0.023 | -0.000 – 0.000 | -0.071 – 0.024 | 0.338 | 0.338 |
| Education | -0.046 | -0.063 | -0.080 – -0.011 | -0.110 – -0.016 | **0.009** | **0.009** |
| Origin | 0.274 | 0.130 | 0.184 – 0.365 | 0.087 – 0.173 | **<0.001** | **<0.001** |
| **Specific Trust * Veracity Discernment** | -0.012 | -0.028 | -0.029 – 0.005 | -0.067 – 0.011 | 0.155 | 0.155 |
| Observations | 1187 | | | | | |
| R^2^ / R^2^ adjusted | 0.467 / 0.464 | | | | | |

*Table S23*. Interaction effects for Vaccine Hesitancy with Institutional Trust and Veracity Discernment as predictors

|  | **Vaccine Hesitancy** | | | | | |
| --- | --- | --- | --- | --- | --- | --- |
| *Predictors* | *Estimates* | *std. Beta* | *CI* | *standardized CI* | *p* | *std. p* |
| *(intercept)* | 2.706 | 0.004 | 0.831 – 4.582 | -0.077 – 0.085 | **0.005** | 0.915 |
| Institutional Trust | -0.017 | -0.074 | -0.310 – 0.277 | -0.159 – 0.011 | 0.911 | 0.087 |
| Veracity Discernment | -0.212 | -0.276 | -0.373 – -0.050 | -0.362 – -0.189 | **0.010** | **<0.001** |
| Age | 0.036 | 0.118 | 0.011 – 0.060 | 0.038 – 0.199 | **0.004** | **0.004** |
| Gender | -0.185 | -0.045 | -0.532 – 0.162 | -0.129 – 0.039 | 0.296 | 0.296 |
| Income | -0.000 | -0.050 | -0.000 – 0.000 | -0.138 – 0.038 | 0.264 | 0.264 |
| Education | -0.092 | -0.048 | -0.257 – 0.073 | -0.135 – 0.039 | 0.276 | 0.276 |
| Origin | 0.915 | 0.191 | 0.532 – 1.298 | 0.111 – 0.271 | **<0.001** | **<0.001** |
| **Institutional Trust * Veracity Discernment** | -0.007 | -0.016 | -0.040 – 0.027 | -0.093 – 0.062 | 0.693 | 0.693 |
| Observations | 530 | | | | | |
| R^2^ / R^2^ adjusted | 0.175 / 0.163 | | | | | |
| *Table S24.* Interaction effects for Vaccine Hesitancy with Specific Trust and Veracity Discernment as predictors | | | | | | |
|  | **Vaccine Hesitancy** | | | | | |
| *Predictors* | *Estimates* | *std. Beta* | *CI* | *standardized CI* | *p* | *std. p* |
| (intercept) | 4.433 | -0.002 | 2.198 – 6.669 | -0.076 – 0.073 | **<0.001** | 0.961 |
| Specific Trust | -0.679 | -0.285 | -1.232 – -0.126 | -0.364 – -0.205 | **0.016** | **<0.001** |
| Veracity Discernment | -0.165 | -0.163 | -0.391 – 0.062 | -0.244 – -0.082 | 0.154 | **<0.001** |
| Age | 0.041 | 0.134 | 0.019 – 0.064 | 0.061 – 0.206 | **<0.001** | **<0.001** |
| Gender | -0.267 | -0.063 | -0.592 – 0.058 | -0.140 – 0.014 | 0.108 | 0.108 |
| Income | -0.000 | -0.064 | -0.000 – 0.000 | -0.142 – 0.015 | 0.110 | 0.110 |
| Education | -0.064 | -0.033 | -0.214 – 0.086 | -0.111 – 0.045 | 0.403 | 0.403 |
| Origin | 0.652 | 0.135 | 0.299 – 1.006 | 0.062 – 0.208 | **<0.001** | **<0.001** |
| **Specific Trust * Veracity Discernment** | 0.005 | 0.005 | -0.058 – 0.068 | -0.060 – 0.071 | 0.878 | 0.878 |
| Observations | 623 | | | | | |
| R^2^ / R^2^ adjusted | 0.209 / 0.199 | | | | | |

**References (Appendices)**

Levi, M., & Stoker, L. (2000). Political trust and trustworthiness. Annual review of political science, 3(1), 475-507.

Maertens, R., Götz, F. M., Golino, H. F., Roozenbeek, J., Schneider, C. R., Kyrychenko, Y., ... & van der Linden, S. (2024). The Misinformation Susceptibility Test (MIST): A psychometrically validated measure of news veracity discernment. Behavior Research Methods, 56(3), 1863-1899.

Newton, K., & Zmerli, S. (2011). Three forms of trust and their association. European Political Science Review, 3(2), 169-200.

J. A. Shoup, “Concerns, attitudes, beliefs, and intentions of parents about Vaccines for their child: Development and evaluation of a survey instrument in an Integrated health care system in colorado,” University of Colorado Denver. (2015).

D. Bussink-Voorend, J.L.A, Hautvast, T. Wiersma, R. Akkermans, M.E.J.L. Hulscher, (2024). Developing a practical tool for measuring parental vaccine hesitancy: A people-centered validation approach in Dutch. *Human Vaccines & Immunotherapeutics*, 21(1). https://doi.org/10.1080/21645515. 2025.2466303

H. Bedford,., Attwell, K., Danchin, M., Marshall, H., Corben, P., & Leask, J. (2018). Vaccine hesitancy, refusal and access barriers: The need for clarity in terminology. Vaccine, 36(44), 6556-6558.

Siegrist, M. (2021). Trust and risk perception: A critical review of the literature. Risk Analysis, 41(3), 480–490. https://doi.org/10.1111/risa.13325
